# Experimental Researches Applied to Physiology and Pathology

**Published:** 1853-08

**Authors:** E. Brown-Séquard

**Affiliations:** Paris


### *Experimental Researches applied to Physiology and Pathology.*

By E. Brown-Séquard, M. D., of Paris.

(Concluded.)

### XXXI.—THE AUDITIVE NERVE IS A NERVOUS CENTRE.

In an anatomical point of view there is no doubt that the auditive nerve is a nervous centre. This is proved by the fact that cells of gray matter are found, not only in the terminal part of the nerve, but also in its trunk, in many animals, according to the researches of Stannius, Corti, Kölliker, and myself.

In a physiological point of view, the fact I have discovered, (see Art. V. p. 21,) viz., that any injury to the acoustic nerve produces turning, is sufficient to prove that it is a nervous centre.

The degree of pain produced by an excitation of this nervous centre appears to be as considerable as that caused by a similar excitation of the trigeminal nerve. I will publish soon an account of the strange effects produced in different parts of the body in consequence of an injury of that nervous centre. I will merely say here that, after such an injury, there are muscles which appear to be slightly paralyzed. Besides, there seems to be a notable hyperæsthesia of the skin everywhere.

Flourens has found that a section of the semi-circular canals in birds and some mammals produces a peculiar disorder in the movements of the head, and, in some cases, turning. He says that the auditive nerve must be considered as composed of two nerves: one going to the semi-circular canals and possessing a peculiar power on the movements of the body, and the other, the vestibular or true auditory nerve. What I have found on frogs is in opposition to these views. A section of the semi-circular canals, in these amphibia, does not produce any effect on the movements of the body, and the slightest excitation of the true auditive nerve is sufficient to produce pain, hyperæsthesia, turning, and other strange effects on many muscles of the body.

I have sometimes seen turning produced after the mere laying bare of the kind of bladder, containing the terminal part of the auditive nerve, in frogs. So slight may be the excitations on that nerve sufficient to produce turning, that very likely turning after the laying bare of that bladder was the result of some slight mechanical injury of the nerve. The rapidity of turning and the smallness of the circle then described are in proportion to the degree of injury to the nerve. When the two auditive nerves are injured, the animal turns on the side most injured. Sometimes, instead of turning, the animals roll around the longitudinal axis of their body; this takes place in very strong animals after the terminal part of the nerve has been entirely crushed.

In frogs deprived of their cerebral lobes, the same effects are produced after injuries of the auditive nerve, as in unmutilated frogs.

### XXXII.—ON APPARENTLY SPONTANEOUS ACTIONS OF THE CONTRACTILE TISSUES OF THE ANIMAL BODY.

All the contractile tissues of the animal body (the muscles of the trunk and limbs, the muscular layers of the digestive canal, the iris, the uterus, the dartos, the cellular tissue, etc.) present, sometimes, apparently spontaneous contractions. I give this name to contractions which are not the result of an external excitation or of an excitation produced by the nervous system on the contractile tissues. These contractions may be permanent or momentary, rhythmical or irregular, slight or very powerful. One of their causes, if not their only cause, appears to be an excitation directly produced on the contractile fibres by the carbonic acid existing in the blood.

1. *Contractions in the muscles of the face after a section of the facial nerve.*—My friend Dr. Martin-Magron and myself have discovered that after the section of one of the facial nerves, on a rabbit, the face becomes very quickly deviated, not on the healthy side, as it is known to be in man, but, strange to say, on the paralysed side. The deviation, very slight at first, increases gradually during one or two weeks, and then it is so considerable that the middle of the lips is at a distance of four, five or six lines from its natural situation. There is an evident state of contraction in all the paralysed muscles. When the animal is excited, or when its respiration is somewhat disturbed or prevented, the paralytic muscles tremble, and sometimes they have rhythmical contractions and relaxations.

The contractions of these muscles may be so considerable that the bones themselves, and, secondarily, the teeth, may be deformed. In one case, on a rabbit which I had kept living twenty-one months after the extirpation of one of the facial nerves, not only the superior and inferior jaws were by far less developed on the paralysed side than on the other, but the anterior part of the superior maxillary bone was deviated towards the paralysed side, so that the middle line of the roof of the mouth was curved and presented a great concavity on the paralysed side and a corresponding convexity on the other.

When the two facial nerves have been divided, there is no deviation, but there is an evident state of contraction in all the paralysed muscles, particularly around the lips.*

When one of the facial nerves is divided on a dog, on a cat, or on a guinea pig, there is generally no deviation on either side. But very frequently there are convulsive movements, and sometimes rhythmical contractions, in the para-

* Dr. Martin-Magron and myself have found that death occurs from inanition in all the species of mammals on which we have divided the two facial nerves. After the operation they cannot swallow: we do not know why.

lyzed side of the face. One of these two kinds of movements always exists in young cats. They are increased, or produced when they do not exist, in dogs and guinea-pigs, almost every time we prevent the animal from breathing freely. Once, on a very vigorous guinea pig, upon which one of the facial nerves had been torn away, I saw alternate contractions and relaxations taking place, without a relapse, for eight or ten days after the operation in the paralysed muscles. After that time, these tremblings appeared only when the circulation and the respiration were rendered very active, or when the respiration was prevented or diminished. In the case of an impaired respiration, the strength and frequency of these movements were in proportion to the degree of asphyxia. During many months, the same phenomena existed in this animal.

I ought to say that in all the experiments above related, the nerve could not have any share in the movements, because, the fifth day after the division, or after the extirpation of a portion of it, the peripheric part had entirely lost its vital property.

In man, as Dugès justly remarks, as long as there is no attempt at movement, voluntary or emotional, the face remains without any deviation, in cases of facial hemiplegia, which have not lasted a long time.

2. *On spontaneous rhythmical or irregular contractions in muscles of animal life, after death.*—It is a very important fact in connection with the theory of the action of the heart, as I will try to prove hereafter, that other muscles, and particularly muscles of animal life, are capable of having rhythmical movements. This fact I have discovered in the following cases:

*a.* After the division of the nerves of the ischiatic and lumbar plexuses, on one side, in mammals, if we suddenly asphyxiate the animal, we see, at first, convulsive movements in the three limbs and in other parts of the body not paralysed. After one, two or three minutes, these movements cease, and there are only some tremblings in the muscles of these parts. The paralysed limb has no movement at all during one or two minutes, after which time, suddenly, contractions in many bundles of muscular fibres partially take place. In the same bundle the contractions sometimes appear to come regularly one after the other. In some cases I have seen, besides these tremblings, movements of the entire limb, consisting of some successive flexions and extensions of the limb, and after these movements had ceased, contractions limited to various bundles of fibres appeared. In these cases the action of the muscles began very late after death, and once, only six minutes after the beginning of asphyxia, which lasted two minutes and a half.

*b.* Nearly the same movements of which I have spoken as existing frequently in the face, during life, in rabbits and guinea-pigs, after the section of the facial nerves, exist always, either during agony or a little after death. They are generally produced by partial contractions and relaxations of the different bundles of fibres of the various muscles. It is rare to see all the bundles composing one muscle contracting together. These phenomena last five, six or eight minutes after the last respiration. There are also such movements in the face during agony and after death, when the nerves have not been cut and when there is no paralysis; but then the movements appear later and do not last so long as in paralysed muscles.

*c.* I have seen in many rabbits apparently spontaneous rhythmical contractions in the respiratory muscles. In about ten rabbits, out of forty or fifty, the following phenomena were very decided; on the others they were slight, and sometimes very slight, but in all cases a part of them always existed. I open the abdominal cavity and expose the bowels to the action of a cold atmosphere, so as to lower the temperature of the animal; after some minutes I make a little opening in one side of the chest, and, at last, after a few minutes more, I open largely one side of the chest. Generally, in such circumstances, the respiratory movements continue to take place with energy. I then take away the sternum and divide the two diaphragmatic nerves. The movement of the diaphragm, nevertheless, continues, and it exists rhythmically together with the movements of the other respiratory muscles. Six, eight or ten minutes afterwards the movements of the diaphragm are still regular, (there are from five to twenty contractions in a minute ;) the intercostal muscles present then only partial contractions. The different bundles of fibres of these muscles contract separately one after the other, but the same bundle has generally regular contractions and relaxations. At that time I destroy the spinal cord, and see that the movements of the diaphragm and of the intercostal muscles are not changed after this operation; they last for nearly a quarter of an hour, and in some cases much longer; their regularity subsists. In the diaphragm, long after the general movement has stopped, there are regular or irregular contractions of many bundles of fibres for one, two, three hours, and sometimes more.

3. *Deviation of limbs produced by a contraction of paralysed muscles.*—In pigeons, after the destruction of all the lumbar part of the spinal cord, the two posterior limbs are completely paralysed. The muscles then are soft, and the different parts of the limbs do not resist at all, when we try to put them in flexion or in extension. But after a few days the paralysed muscles become harder, and after a few weeks there is an evident state of contraction in them. The limb is generally kept in a state of extension, and deviated on one side or the other. The deviation becomes considerable after some months.

Very likely it is owing to the same cause that club-foot and other deviations are produced in embryos, after a destruction or an absence of development of the spinal cord.

4. *Rhythmical movements in the eye of the Ink-fish.* (Loligo sepia, L.)—The ciliary muscle so well described by Dr. W. Clay Wallace, of New York, in the eyes of superior animals, is strongly developed in the ink-fish. After an eye of this mollusc has been separated from the body, I have sometimes found very singular and perfectly rhythmical movements produced by the ciliary muscle. These movements consisted in alternative contractions and relaxations of some parts of that muscle. At every contraction a notable depression was produced in one portion of a zone corresponding to the circumference of the cornea.* In one case I have found four times in fifteen minutes the same rhythm existing in one part of the ciliary muscle. At each of these four examinations I have found sixteen contractions in one minute.

5. *Spontaneous Contractions of the Uterus.*—I have seen hundreds of times the uterus or its cornua, full or empty, contracting to appearance spontaneously, after the death of rabbits and other animals, at a time when the spinal cord had entirely lost,

* The eyes had not been opened.

not only its reflex power, but also the power of acting on muscles when directly excited by galvanism, by warmth or mechanically.*

I have also seen movements taking place in the uterus and in its cornua, in recently dead animals, the spinal cord of which I had destroyed in all its length. The same movements I have found after I had taken out from the abdomen of a living animal the whole uterine apparatus. I have found sometimes that after I had put a ligature around the trachea of guinea pigs, which were at the end of gestation, parturition took place and was produced by three causes: 1st, a direct excitation of the spinal cord by the venous blood; 2d, a direct excitation of the uterus by that blood; 3d, a reflex action of the spinal cord. In two cases I have seen delivery taking place after the action of one only of these three cases, namely, the direct influence of black blood on the uterus of the Guinea pigs, the spinal cord of which I had destroyed from the sixth costal vertebra to the sacrum. The more complete and sudden is the asphyxia, in a rabbit or a Guinea pig, during labor, the more certain will the delivery take place.

Dr. Tyler Smith speaks of a peristaltic action of the uterus, which may expel the child when the mother has died during labor, undelivered. He has not attempted at all to explain that con-

*Dr. Tyler Smith, in his very original book on Parturition, (London, 1849, p. 40,) says that "a slow reflex action of the uterus may possibly continue long after the rhythmic respiratory actions have ceased; as long, indeed, as the body retains its warmth." There is a great error in these lines, about the relation between the warmth of the body and reflex action. We may observe reflex actions even in animals that have lost 10, 12 or 15° Cents., (18, 22 or 27° Fahr.,) of their temperature, and, in certain circumstances, these actions may be, then, more powerful than if the temperature of the body was normal. For instance, if we decapitate an animal after having put a ligature around the carotid and vertebral arteries, we find, when pulmonary insufflation is made carefully, that two important phenomena take place—one is a gradual rapid loss of temperature, if the atmosphere is cold, (this is the well known fact discovered by Sir B. Brodie,) and the other is a gradual and considerable increase of the reflex faculty. It has been in such cases that I have found the greatest degrees of reflex power in mammals. The nervous power accumulates to such an extent in the spinal cord, that if we pinch the skin in any part of the body, but more particularly on the chest and on the anterior limbs, a reflex respiratory movement takes place.

traction, *i. e.* to find out its cause and the circumstances which favor or are opposed to its existence; besides, he has not demonstrated that the peristaltic contraction is entirely independent of the nervous system.

6. *Spontaneous rhythmical movements in the crop and œsophagus of pigeons and other birds.*—I have found that if the crop of a bird, and more particularly of a pigeon, is opened during digestion, some rhythmical movements are frequently seen in it and in the œsophagus. Ordinarily these movements are perfectly regular. They begin in the upper part of the crop, and are propagated from there to the œsophagus. If the animal is asphyxiated, these contractions become very energetic. Their ordinary number, in a minute, varies from ten to twenty.

I have ascertained that these rhythmical movements take place as well in a crop and œsophagus separated from the animal, as in these same parts left *in situ.* Therefore, the nervous centres are not the source from which originates the excitation which acts on the muscular fibres to put them in contraction.

7. *Spontaneous movements in limbs of persons who have died of cholera.*—It is known that after death by cholera, the whole body, and more particularly the limbs, have sometimes very considerable movements. In some cases I have seen alternative movements of flexion and extension of the arms or of the legs, even three hours after the cessation of the beatings of the heart. Physicians who know how quickly after death the nervous system loses its vital powers, will admit easily that these movements cannot be the result of an action of that system. I have ascertained on more than sixty bodies of men who died of cholera, or of various other diseases, that a short time before, or a very short time after the cessation of the beatings of the heart, no reflex action was produced by the tickling of the sole of the foot. The greatest duration of reflex action that I have observed after death has been in a case of cerebral apoplexy. It has lasted thirteen minutes after the last breathing, and about eight minutes after the last beating of the heart. Dr. Bennet Dowler has recorded many curious facts (observed in cases of death from yellow-fever, cholera, etc.,) from which he concludes also that the movements taking place in the limbs are not reflex actions.

I have found that, in general, the more sudden and complete has been the asphyxia before death, by cholera, the more the limbs are moved after death. I have found also that it is in patients who have died during the algid period that these movements are ordinarily found.

These facts, as I will show hereafter, appear to prove that these movements, like the other movements, of which I have previously spoken, are excited by carbonic acid alone, or together with the poison of cholera.

8. *Spontaneous contractions of the bowels, the bladder, the iris and other parts of the body.*—It is known that frequently at the time of death, many of the contractile tissues of the body are put into contraction. I can go farther and say that it is so with *all* the contractile tissues; and that, contrary to the general opinion, a nervous action is not necessary for these contractions.

There are contractions in all the following organs or tissues during agony and after death: 1, the muscles of animal life; 2, the sphincter of the anus; 3, the respiratory muscles; 4, the iris; 5, the digestive canal (in all its length); 6, the urinary bladder; 7, the uterus; 8, the scrotum, (dartos); 9, the gall-bladder; 10, the ureters; 11, the seminal vesicles; 12, the bronchial tubes; 13, the skin; 14, the blood-vessels; 15, the lymphatics; 16, the cilia.

As to the skin, in many cases the so-called *goose-flesh* (cutis anserina) takes place a little before or little after death, although the body has not yet become cold. I have seen it very strongly marked on the inferior limbs of a paraplegic who died of a softening of the dorso-lumbar part of the spinal cord. It results from this fact that the cellular tissue is able to contract from the same cause which produces contractions at the time of death, in muscular tissues—that is, very likely, carbonic acid.*

* Kolliker has recently discovered fibro-muscular cells—that is, muscular fibres of organic life—in the skin, and he maintains that the *cutis anserina* is produced by these fibres, and not by the cellular fibres. I have published facts which, I think, prove conclusively that the contractions in the skin are in a great measure performed by the cellular tissue. (See Comptes Rendus de la Soc. de Biologie, 1849, t. i. pp. 134 et 157, et 1850, t. ii. p. 132.) Since that time, I have found that in some cartilaginous fishes, in which the iris does not contain any muscular fibre, and is composed of cellular tissue, this membrane may be the seat of considerable contractions; so that I consider it as perfectly certain that the cellular tissue (at least in some organs) is contractile.

Besides, I have found contractions of the cellular tissue of the skin of the face, in animals killed by asphyxia, and on which the facial nerve had been divided for many days or weeks.

In the same man who had a paraplegia, and of whom I have just spoken, I saw very strong contractions in the dartos, during agony.

In animals suddenly asphyxiated, after the destruction of the dorso-lumbar part of the spinal cord, the seminal vesicles sometimes contract, and a slow ejaculation takes place, although there is no erection.

In the sphincter of the anus, when it is paralysed, there are only slight contractions, but they are evident.

The urinary bladder, during agony or after death, sometimes contracts so much, even when it is paralysed, that all the urine it contains is expelled.

The ureters present very strong contractions, in animals recently killed by asphyxia, and these contractions in some cases are rhythmical. The same movements are seen when all the urinary apparatus is *in situ*, and when it has been removed from the abdomen, and therefore separated from the nervous centres. The contraction begins at the kidney and thence is very quickly propagated all along the ureters to their termination in the bladder. Among the contractile tissues, that of the ureters is one of the most irritable.

Bidder and Schmidt, of Dorpat, have recently found that after the division of the two pneumogastric nerves, there is more carbonic acid expelled by the lungs than usual. This fact is very important, because if the theory, which I am about to propose, be true, we ought to see a contraction produced in the bronchial tubes, in consequence of the unusual amount of carbonic acid that they contain. Now, such a contraction certainly exists then, and it is it which causes the well-known difficulty in the expansion of the chest, which exists in that case.

In the eyes, even when they are paralysed by the section of the three nerves of the iris, (the third pair, the sympathetic, in the neck, and the ophthalmic nerve,) the pupil may, at first, contract and afterwards dilate very much.

The lymphatics and the thoracic duct contract very much after death. I have, sometimes, in cases where these vessels were dilated by chyle, introduced a glass tube, two lines in diameter, into the thoracic duct, and I have seen the liquid ascend into the tube, and in one case run out, although the tube was five inches high.

The cilia are known to have movements independent of the nervous system.

The gall-bladder contracts little and slowly, but evidently, after death, even when it has been, with the liver, removed from the abdomen and separated from the nervous centres.

The choledoch duct and the pancreatic duct, as my friend Cl. Bernard has discovered, have rhythmical contractions during life, in birds. I have found these movements perfectly regular after I had removed all the viscera from the abdomen. Therefore the cause of these rhythmical contractions is not in the nervous centres.

The bowels have considerable contractions during agony and after death; and I will prove hereafter that the cause of these movements is not the influence of cold, or that of air, when they are exposed to the atmosphere. Nurses, in France, are in the habit of judging that death has positively taken place, when, after the cessation of breathing, they see urine and fæcal matters expelled. This expulsion depends upon the contractions then taking place in the bladder and in the bowels.

9. *Causes of the apparently spontaneous contractions during life and after death.*—All the contractions of which I have spoken, appear to me to be produced by an excitation made upon the contractile tissues by a substance existing in the blood, and the quantity of which becomes much increased during asphyxia. The relations between these contractions and asphyxia are evident. A great many of them do not exist unless asphyxia exists, and their energy is always in proportion to the degree of asphyxia.

I believe that the substance in the blood which has that power is the carbonic acid. In admitting this opinion we can easily explain all the phenomena.

There are certain contractions which take place in muscles of animal life, after death, and which have quite another cause. In the cold seasons, it is not uncommon to find, in limbs of frogs, when we separate them from the body, apparently spontaneous contractions, lasting sometimes for half an hour or even more; but these contractions have begun when we have cut the nerves, and they continue on account of galvanic discharges which accompany them. The fact that they begin after the excitation of a nerve, is sufficient to show that they are not like the other contractions, of which I have previously spoken.

Some of the facts I have related may appear to be distinct from the others. So, for instance, contraction taking place in paralysed muscles of the face or of the limbs in living animals, might be considered as quite different from the contractions existing after death. I think that they originate from the same cause, viz., an excitation by carbonic acid. A muscle may be moved or not be moved by an excitant. If the degree of irritability is greater in one case than in another, we may see the same amount of excitation produce a movement in the first case, and not in the second. If the amount of excitation increases, then we may see both muscles moved, but the most irritable more than the other. This is sufficient to explain why the paralysed muscles may be moved by the carbonic acid existing in the blood during life, while the muscles that are not paralysed are not moved. I have found that the degree of irritability increases, during a certain time after paralysis, in the muscles of animal life. Their irritability being augmented, they are excited sufficiently to contract, by a quantity of carbonic acid which is not sufficient to act on the other muscles.

The following facts and reasonings will, I believe, prove that, at least in the bowels, black blood, very likely by its carbonic acid, may excite powerful movements. It is known that when we open the abdomen of an animal immediately, or a short time, after death, we generally see considerable movements in the bowels. These movements have been attributed to the action of air, or to that of cold, on the bowels. This is not a right view. A sudden exposure to a cold atmosphere may, possibly, produce contractions in the bowels; but certainly cold is not the ordinary cause of these movements. At first, they may exist in a warm atmosphere, and then they appear to be more rapid than in a cold atmosphere. Besides, the bowels may be exposed to a cold atmosphere, and remain motionless, although they have their entire irritability. As to atmospheric air, it is not able to excite a movement in the bowels. If we open the abdomen of a living animal, in avoiding to excite mechanically the bowels, and in allowing the animal to breathe freely, we may for a long time see no other movement in the bowels, except, sometimes, slight regular and natural peristaltic motions, depending on digestion, and limited to some small parts of the bowels. The animal must be kept on his back, and we must avoid touching the bowels, because a slight contact is sufficient to produce movement. Now, if we prevent the animal from breathing, we see, after ten, fifteen, or twenty seconds, very violent, sudden, and rapid contractions taking place in all parts of the intestine, from the stomach to the rectum, but much more in the small intestine than elsewhere. These movements are quite different from the digestive peristaltic movements. If the animal is allowed to breathe again, and freely, the movements diminish gradually, and disappear almost entirely after a few minutes. Then, if we prevent it again to breathe, we see the movements produced again. This experiment may be repeated many times, with the same result, on the same animal.

We are certainly entitled to conclude that there is an exciting cause of contractions, developed during asphyxia, and that it is neither the cold nor the atmospheric air which produces in all cases the movements of the bowels after the opening of the abdomen. We may draw the same conclusions from another experiment. If we put a tie around the trachea of a living animal, immediately after expiration, we may see and feel violent movements taking place in the bowels, although the abdomen is not opened. It is in consequence of such movements that there is an expulsion of fæcal matters, after death, in man. The urine may be also expelled in these cases, in man and in animals, and this expulsion takes place because the bladder contracts, and not, as it is generally admitted, because the *sphincter vesicæ* becomes relaxed.

Some physiologists have considered the cessation of the circulation of the blood in the bowels as the cause of their movements, after death, and they relate as a proof the fact that the section of one of the arteries going to a part of the intestines, is followed by contractions in the parts thus deprived of circulation. But nothing is explained by saying that the cause of the contraction is in the absence of circulation. As contractions require an excitation to be produced, what is the exciting cause when the blood does not circulate? After the section of an artery there is blood remaining in the capillaries, and that blood, after a short time, becomes very rich in carbonic acid, and then, if my theory is right, contractions ought to be produced. The result of the section of one of the arteries is, therefore, in accordance with my theory.

Other facts may be adduced proving the influence of black blood and carbonic acid on the bowels.

If black blood is injected in the arteries of the small intestine when its irritability is much diminished, movements are almost immediately produced, but they do not last long. On the contrary, if red blood is injected, movements do not appear immediately, and they are very strong and last long. This action of red blood may be easily understood: it increases the irritability of the muscular layer of the bowels, as it does for that of the muscles of animal life, and when it has been changed into black blood, it excites the muscular tissue and produces contraction. The strength and the long duration of the contraction in this case depend on the increase of irritability. When, as in the above experiment, black blood (containing a great quantity of carbonic acid, on account of the constant formation of that gas in blood deprived of the contact of atmospheric air) is injected, the irritability is not sensibly increased, but the excitation is considerable and there is an almost immediate effect.

If air is injected in the arteries of the bowels, soon after the death of the animal, a part of the blood it contains is expelled, and we find that the movements do not last as long as if the blood had not been removed.

When an animal is killed by hæmorrhage, the intestine, as well as all the other organs, contains more blood than usual, and then its movements are not so strong, and last less than they do generally.

When in a recently asphyxiated animal the arteries and veins of a part of the bowels are divided, the movements of that part become less strong and last less than those of the other parts of the intestine.

When the bowels of a recently asphyxiated animal are put under a receiver containing carbonic acid, their movements are very much increased, but they do not last so long as when they are in the atmosphere.

When they are put under a receiver containing hydrogen, their movements are very quickly diminished in strength, and they last still less than when exposed to carbonic acid.

When they are put in oxygen, their movements diminish a little at first and soon after become stronger, and they last much longer than usual.

As a general conclusion about the apparently spontaneous contractions which I have described as taking place in paralyzed muscles during life or after death, I will say that it seems that black blood by its carbonic acid is the cause of these contractions. When the nervous centres are still united with the contractile tissues, we see, during agony or after death, stronger movements generally than when they are separated. The action of black blood on the nervous centres may be very great. I have found that the spinal cord, when separated from the encephalon, may be strongly excited by black blood. If an animal is asphyxiated after a transversal and complete division of its spinal marrow in the dorsal region, we see convulsions taking place in the posterior limbs, and they are nearly as strong as when the nervous centers have not been injured. The excitation on the spinal marrow is considerable enough to produce an erection of the penis.*

XXXIV.—ON THE CAUSE OF THE BEATINGS OF THE HEART.

The cause of the rhythmical movements of the heart has been heretofore unknown. I believe I have discovered it.

Before exposing my theory and the facts upon which it is grounded, I will show that the theories put forward until now are not correct.

There are three theories only which are worthy of examina-

* Almost all, if not all, the secretions of the body are increased during asphyxia: bile, (as shown by Professor Bouisson,) saliva, tears, gastric, pancreatic and intestinal juices, and also liver-sugar, etc., are produced in greater quantity then than usual. I believe that this increase results from the excitation of the nervous system, and, in some measure, perhaps, from a direct action of black blood on the capillaries of the glands. The urinary secretion may also be changed in asphyxia, and not only then the urine may contain sugar, as Alvaro Reynoso has found, but also albumen.

tion: 1st, that of Haller; 2d, that of Carpenter; 3d, that of Budge, Schiff, and others.

Haller has been very near the truth in admitting that the beatings of the heart were excited by the blood. His error has been an *error loci.* He thought that the blood acted in the cavities of the heart. It is not so; and it is known that the heart may continue to beat after all the blood has been drawn out of its cavities.

The doctrine of Carpenter* is a very simple and remarkable one. He believes that the muscular fibres may act without having been excited. A muscle, says he, may be compared to the electric jar, and become so *charged* with *motility,* (or motor force,) as to execute spontaneous contractions; and elsewhere, "It is not very difficult to conceive that the ordinary rhythmical movements of the heart may be due to a simple excess of this motility, which is continually being supplied by the nutritive operations, and is as constantly discharging itself in contractile action." Carpenter believes that the reason for which the heart presents spontaneous contractions while the other muscles do not, (at least ordinarily,) is, that there is a higher degree of motility in the heart. He considers as very important the facts I have discovered, that many other muscles besides the heart may present rhythmical movements. He thinks that these facts show there is a tendency to rhythmical movements in the muscles themselves, altogether independent of the excitement to action which they receive through the nervous system.

The best ground for the hypothesis of Carpenter is that, according to him, the heart continues to beat, although it is not exposed to any excitation in certain circumstances. He says: "When every source of excitement is excluded, we cannot but perceive that these actions take place with a *spontaniety* which can scarcely be accounted for in any other way than by considering them as expressions of the vital activity of the component cells of these forms of muscular tissue, which manifests itself in this mode, when the developmental life of the cell has attained its maturity. And this view is strikingly confirmed by what we know of the origin and termination of these movements.

* See his *Principles of Human Physiology, American edition,* by F. G. Smith. Philadelphia, 1853. pp. 130 to 132, 319, 325, and 471–72.

For the action of the heart commences when, as yet, its contractile parietes consist but of an assemblage of ordinary-looking cells, no proper muscular tissue being evolved, and no nervous system being yet developed, from which the stimulus to the movement can proceed; and *it is impossible to assign any other cause for the movement under such circumstances*, than the attributes inherent in the tissues which perform it."

The first thing to be said against the view of Carpenter is, that his hypothesis is not necessary; because *it is possible to assign another cause for the movement of the heart under the circumstances* he speaks of. This will be proved hereafter.

The doctrine of Carpenter implies, that the degree of irritability (motility, motor force, contractility,—never mind the name) is greater in the heart than in the other muscles which have no spontaneous action. This is not the case. The degree of irritability, as judged by its duration after death, is generally greater in the muscle of animal life, than in the heart. The accepted sentence of Haller, *Cor ultimum moriens*, generally, is not true.

If Carpenter was right, we should see, during life, the apparently spontaneous contractions which take place in all the contractile tissues after death; because their irritability is at a higher degree in the first, than in the second case. Besides, we should not see oxygen, or red blood, diminish the frequency of the beatings of the heart; and black blood, or carbonic acid, increase that frequency.

An experiment, consisting in the research of the influence of *vacuo* on the heart, has been made by Tiedemann and by Dr. S. W. Mitchell, and Dr. T. H. Bache, (see Dunglison's Physiol., vol. ii. p. 150.) It seems to me that the result of this experiment is in complete opposition to the doctrine of Carpenter. These experimenters have found that the beatings of a heart were speedily brought to a stand by the exhaustion of the air, and that they were renewed when it was re-admitted. If the view of the eminent British physiologist was right, we ought to see the heart continue to beat in vacuo about the same length of time as it would in hydrogen or nitrogen, because its irritability cannot be suddenly diminished enough by the exhaustion of the air. In these gases the heart of a mammal may beat for five or ten minutes or more, and the right auricle may beat for hours; and the heart of a frog may beat for one day. It is much more to account for the stopping of the heart's action in admitting that the excitant of that action is removed during the exhaustion of the air. John Reid had found that the heart of a frog had continued to beat *in vacuo*, but how long he does not say.*

I will relate hereafter many experiments of mine which are in opposition to the theory of Carpenter.

It is one of the most important questions in physiology, whether the nervous centres, the nerves, and the contractile tissues are able to act without stimulation. This question has not been yet entirely treated by any physiologist. I propose publishing a special paper on the subject. I will merely say here that there may be apparently spontaneous actions in the spinal cord, as well as in the muscles. For instance, very frequently, in a frog, after the removal of the brain and the medulla oblongata, we may see strong movements apparently spontaneous, but when we know that the slightest excitation of the skin, or of any other very sensitive part, may excite the spinal cord, and produce a reflex action, we are authorised to consider all the movements taking place as reflex actions. An excitation may have come to the spinal marrow from the bladder, from the bowels, from the lungs, (in which worms are almost always found in the cold seasons, i. e. at the time these phenomena are generally observed,) etc.

As to the spontaneity of action in muscles, I have tried to prove in a preceding article that it is a mere and false appearance.† I will prove hereafter that the cause of the apparently

* Art. Heart, in Todd's Cyclop., vol. ii. p. 611. J. Reid says in the same page, "We ought to be more cautious in admitting the existence of this innate moving power, since it is in opposition to a well known law in the animal economy, that though the various tissues of an organised body are endowed with certain vital properties, yet the application of certain external and internal stimuli is necessary to produce their manifestations of activity. In fact it is from the action and reaction of these tissues and excitants upon each other that the phenomena of life result."

† Carpenter says that the action of the uterus, as it shows itself, "not merely in the final parturient effort, but in local contractions that frequently occur during the latter months of gestation, (simulating the movements of the fœtus,) are more satisfactorily accounted for by considering them as a discharge of accumulated power, than in any other mode." I will try to spontaneous contractions of the heart, is the same as that of the like contractions in other contractile tissues.

The physiologists who maintain that the beatings of the heart depend on the nervous system, appear to me to be greatly mistaken. They make a confusion between two things, greatly distinct, one from the other: they conclude from the fact that the nervous system is able to act on the heart, that its influence is necessary. It is the same kind of mistake which is so frequently made as to the influence of the nervous system on nutrition, on secretions, and on animal heat; because that system is able to act upon these functions, it is concluded that its influence is necessary.

The first argument to be adduced against the writers who admit, as necessary, the influence of the nervous system on the heart, is, that they change only the ground of the difficulty in doing so. Instead of having to explain why the heart acts rhythprove elsewhere that for the uterus, as well as for any other contractile tisue, there is no spontaneous action. The uterus, in pregnancy, becomes more and more irritable every day, and when its irritability has arrived at a very high degree, then the slight excitation produced by the carbonic acid normally contained in the blood is sufficient to put it in action. When the contractions have begun, they are very much increased by a reflex action. Every contraction is accompanied by a galvanic discharge on the nerves in the neighborhood of the muscular fibres which contract, and the sensitive nerves being thus excited, it results, 1st, that a pain is felt, the degree of which is in proportion to the degree of any contraction, and therefore with the degree of galvanic discharge;* 2d, that the spinal marrow is excited, and produces reflex movements in the uterus. Now, the more these reflex contractions are energetic, the more they are induced to take place again, on account of the galvanic discharge which accompanies them. So that there would be a constant increase in the intensity of the contractions if there were not four limits to them. 1st, there is no galvanic discharge when the muscular fibres *are contracted;* it is only at the time they *are contracting* that this discharge takes place; 2d, the primitive cause of contraction, the excitation of the muscular tissue, by carbonic acid, diminishes much during the contraction, because the caliber of the small blood-vessels is much diminished, and the blood expelled from them; 3d, every contraction of the uterus diminishes the degree of its irritability; 4th, the reflex power of the spinal cord becomes exhausted, or at least diminished.

* See, on this subject, my paper in the Comptes rendus de la Société de Bologne, en. 1850, t. ii. p. 172.

mically, they have to explain why the nervous system acts rhythmically on the heart. Not only they have not explained this rhythmic action of the nervous system, but, as far as I know, they appear not to have been aware that this was to be explained.

The second reason I will mention, is the fact, so well estabilsed by my friend Professor Lebert, that, in embryos, the heart beats when it is merely composed of cells, and when the nervous system has not yet appeared.

A third reason is, that, either in monsters, or in animals operated on by physiologists, there has been a long persistence of the beatings of the heart when a part of the cerebro-spinal centre did not exist, or had been removed. Any part may be in that case, even the medulla oblongata, as I have discovered. (See Art. xvi. p. 40.)

In opposition to the idea that the beatings of the heart depend on the microscopical ganglia existing in that organ, I will say, that, besides the fact that the heart beats in embryos before the nervous system exists, and besides the improbability that such a small amount of nervous matter should have so great a power, there are two good reasons against this strange theory:—1. There have been found no ganglia, large or microscopical, in the auricles, in the sinuses of the pulmonary veins, or in those veins. All these parts, nevertheless, may continue to beat a long while, (even for hours,) after they have been separated from the ventricles where are the microscopic ganglia. 2. Rhythmical movements may exist in a great many other muscular parts of the body, where there is no microscopical ganglion, and where these parts have ceased to be under the influence of the nervous centres.

The three theories which I have examined being unable to explain the beatings of the heart, I will now expose my theory, and discuss the three following questions:—1. What is the excitant which puts the heart in action? 2. Does that excitant act rhythmically? 3. Does that excitant act together directly on the muscular fibres of the heart, and on the nervous system; or does it act only on the muscular fibres?

After having solved these three questions, I will examine the objections which might be made to the doctrine I propose.

1. *What is the excitant which puts the heart in action?*

I believe that the beatings of the heart are excited by a principle existing in the blood, and that carbonic acid is that principle. This view is grounded on the following facts:

*a.* When we prevent a warm-blooded animal from breathing, the beatings of the heart become more frequent than before, for about one or two minutes. It is not on account of the emotion alone that it is so, because the same effect is produced when we asphyxiate suddenly an animal which has entirely lost his power of having emotions, in consequence of the action of chloroform.

*b.* Many times I have found, on myself and on one of my friends, that the beatings of the heart are rendered more active during asphyxia. We hold our breath for about three quarters of a minute, and during the last fifteen seconds the heart beats from two to four (in one case five) minutes more than when the respiration was free. We have made the experiment in the sitting position, avoiding any movement of the body in all the cases.

*c.* John Reid has discovered that when any hemadynamometer is put in the femoral artery of a dog, the mercury rises in the instrument if the animal is asphyxiated, and about one minute after the respiration has been stopped. The same result has been obtained in twenty experiments. It seems to me that this fact proves that the contractions of the heart become more energetic during asphyxia. John Reid attributes the result he has obtained to some difficulty that black blood seems to have in passing through the capillaries of the different parts of the body. I do not deny that there is such a difficulty; but I think that the great reason of the ascension of mercury in the hemadynamometer is, the increase in the force of the heart. A simple experiment proves that I am right. I adapt the hemadynamometer to the aorta in the abdominal cavity, and then I open quickly the chest, and I put a ligature to the brachial and carotid arteries. About three quarters of a minute after opening the chest, and about half a minute after the ligature has been put on the arteries of the head and arms, the mercury rises notably in the instrument; sometimes the elevation is as considerable as two inches. It results from this experiment, that the heart beats more strongly in asphyxia about one minute after its beginning.

*d.* Woodall, a most intelligent and accurate observer, says Dr. Martin Paine, (see Med. and Physiol. Comment., t. ii. p. 49,)

states, that the best remedy for syncope is to obstruct respiration entirely by momentarily confining the nose and mouth. If this be true, it is in perfect accordance with my view, that, during asphyxia, the normal cause of the beating of the heart increases in the blood.

*e.* If a frog is put under a receiver containing pure oxygen, at a temperature of 40 or 50° Fahr. (4, 5, or 10 Cent.) after its heart has been laid bare and its central nervous system destroyed, we see the heart beat for a very long time, (one, two, or three days.) On the contrary, if, at the same temperature, another frog, deprived also of the central nervous system, is put in carbonic acid gas, the heart beats very quickly at first, but it soon ceases to beat, (in one or two hours only, sometimes, and for the most about half a day.)

*f.* All the causes which increase the formation of carbonic acid gas in the body, increases the frequency of beatings of the heart.

*g.* If we inject the serum of blood into the arteries of the heart, so as to expel as completely as possible the blood contained in the capillaries of this organ, and if then we remove the blood from the cavities of the heart, we find that its beatings are, at once, almost entirely suspended, and that they are completely stopped in a very short time, (from one to eight minutes.) The muscular irritability is not destroyed in this organ; it does not beat because its excitant has been removed.

*h.* I have found that when the heart of a young animal is put in hydrogen, its beatings hardly change at first, but they stop in a very short time. When it is put in carbonic acid gas, its beatings are, at first, increased in frequency and strength; but they very soon are stopped. When it is put in oxygen, its beatings are slowly increased in frequency and strength, and they last very long.

*i.* On newly-born cats and dogs, before the occlusion of the *ductus arteriosus*, I open the chest and put a ligature on the arteries going to the head and fore-limbs, and on the aorta immediately after the origin of the ductus arteriosus. Then the blood, expelled from the right ventricle, is sent to the lungs, from which it comes to the left auricle, and afterwards to the left ventricle. From there it is sent into the only part of the aorta remaining accessible, and thence it goes into the cardiac arteries, and into the pulmonary artery, through the ductus arteriosus, (a direction which is the reverse of the normal direction in that duct.) By the cardiac veins the blood arrives again in the right side of the heart. The circulation from the heart to the lungs, and *vice versa*, continues very well. I have found, that if hydrogen is insufflated into the lungs, the beatings of the heart are not much changed at first, but they go on diminishing, and they disappear in a short time. When an injection is made with carbonic acid, the beatings of the heart are quickly increased in frequency and strength; but they are stopped after a short time. When oxygen is insufflated, the beatings of the heart become slowly more frequent, and they remain quick and strong for a long time. (I have once, by such insufflation of oxygen, maintained beating for eleven hours in the heart of a young cat.)

I believe that these facts prove that black blood, by its carbonic acid, is an excitant of the beatings of the heart. If, now, we adduce to these facts all those I have related in a preceding article, on the apparently spontaneous contractions in all the contractile tissues of the body, we shall have a very considerable number of facts, proving that, during asphyxia, there is an accumulation in the blood of the principle which causes these contractions. I believe that it is almost impossible to deny that this principle is the carbonic acid gas.

Before trying to show that what takes place in asphyxia in the heart is only an exaggeration of what normally exists in that organ, I will treat the two remaining of the three questions I have announced I would endeavor to solve, as regards the excitant of the heart's action.

2. *Why does that excitant act rhythmically?*

I believe it is easy to explain why the agent of excitation of the heart* produces rhythmical contractions. I will suppose, first, that the action is permanent. A part of the heart, ventricles, or auricles, being dilated, receives an excitation in all its fibres simultaneously, and a contraction is produced. But, according to the well-known law of Schwann, the exciting cause which is able to give the impulse when the muscular fibres are long, is not able

* What I will say here for the heart, might be said for all the contractile tissues, presenting apparently spontaneous rhythmical contractions, as the cilia, for instance.

to maintain the contraction when the fibres have been shortened. Then, on account of this insufficiency of power of the cause of the contraction, a dilatation ensues. We may present the fact in other words, and say that the resistance to the contraction originating from the displacement of the constitutive matter of the contractile tissues, increases in proportion to the shortening of the fibres; and that after the fibres have contracted under the impulse of the exciting cause, although this cause continues to act, a dilatation is produced by the force belonging to that resistance, which is nothing but elasticity. If the cause of the contraction of the heart was a considerable one, then we should see a permanent contraction; and it is so when we apply galvanism—the elasticity, then, is not powerful enough to produce dilatation. On the contrary, with a weak exciting cause, like carbonic acid, the result ought to be different. When that cause has more power, as in asphyxia, the shortening of the fibres takes place quicker, and is more considerable; and even then it is not sufficient to maintain contraction, the tendency to dilatation being also increased.

I ought to say, that the excitant cause of the contractions is not always at the same degree of power. The small blood-vessels and the capillaries being compressed during the muscular contractions, there is a diminution of excitation during that time. This should be sufficient to explain the alternate contractions and dilatations. But such a diminution in the caliber ought to be very little, if even it exists in certain organs, (the heart when composed of cells, for instance.)

I come now to the third question about the excitant of the heart—

3. *Does that excitant act together on the muscular fibres and on the nerves of the heart, or does it act only on the muscular fibres?*

I believe it ought to act also on the nerves; but I cannot prove it otherwise than by saying, that all the agents of excitation that we know to act on the muscular fibres, are able to act on the nerves.

There are many things to be said besides the above facts and reasoning, to prove the truth of the doctrine I propose. I will expose some of them.

The following question might be made:

How is it that the heart is the only muscle containing striated fibres, which presents normally rhythmical movements?

The answer to this question appears to be very simple. The intensity of the stimuli, the degree of irritability, and the resistance which a muscle has to overcome when it contracts, are three elements which we ought not to lose sight of when we examine the difference of contractions between two muscles. Suppose the heart possessing the same degree of irritability as another muscle: if the stimulus is the same, and the resistance the same also, for the heart and for the other muscle, there will be the same effects. But if the stimulus is more considerable in the heart than in the other muscle, and if the resistance to be overcome is less for the heart, then with the same degree of irritability in both parts, and even with less irritability in the heart than in the other muscle, we will see a movement in the heart, and not in that other muscle. Now a simple examination of the vessels of the heart, proves that they contain more blood, and consequently more stimulus, than the other striated muscles. Besides, as the heart is not inserted into heavy bones to be moved, it has less resistance to overcome when it has not to circulate the blood, as after death, or when it is out of the chest, than the muscles of animal life. Some muscles in the face and the diaphragm, being almost without an external resistance, when their contractions do not go so far, it results that they are moved much more easily after death, than the muscles of the limbs. In consequence of these views, I believe that, although there is in the blood-vessels of all the muscles of the body a principle which is an exciting cause of contractions, there are no contractions produced, because the quantity of that principle is not sufficient, or because the resistance to contractions in many muscles is greater than in the heart.

I must, in conclusion, say, that I do not advance my theory of the rhythmical movements as perfectly proved. I believe it is true, and that there are a great many facts which appear positively to prove it. What I can assert is, that it is by far much more in harmony with all the known facts, than the other theories.

